# A Deep Catalog of Autosomal Single Nucleotide Variation in the Pig

**DOI:** 10.1371/journal.pone.0118867

**Published:** 2015-03-19

**Authors:** Erica Bianco, Bruno Nevado, Sebastián E. Ramos-Onsins, Miguel Pérez-Enciso

**Affiliations:** 1 Centre for Research in Agricultural Genomics (CRAG), CSIC-IRTA-UAB-UB Consortium, Bellaterra, Spain; 2 Universitat Autònoma de Barcelona, Department of Animal Science, Bellaterra, Spain; 3 Institut Català de Recerca I Estudis Avançats (ICREA), Carrer de Lluís Companys 23, Barcelona, Spain; Kunming Institute of Zoology, Chinese Academy of Sciences, CHINA

## Abstract

A comprehensive catalog of variability in a given species is useful for many important purposes, e.g., designing high density arrays or pinpointing potential mutations of economic or physiological interest. Here we provide a genomewide, worldwide catalog of single nucleotide variants by simultaneously analyzing the shotgun sequence of 128 pigs and five suid outgroups. Despite the high SNP missing rate of some individuals (up to 88%), we retrieved over 48 million high quality variants. Of them, we were able to assess the ancestral allele of more than 39M biallelic SNPs. We found SNPs in 21,455 out of the 25,322 annotated genes in pig assembly 10.2. The annotation showed that more than 40% of the variants were novel variants, not present in dbSNP. Surprisingly, we found a large variability in transition / transversion rate along the genome, which is very well explained (R^2^=0.79) primarily by genome differences in in CpG content and recombination rate. The number of SNPs per window also varied but was less dependent of known factors such as gene density, missing rate or recombination (R^2^=0.48). When we divided the samples in four groups, Asian wild boar (ASWB), Asian domestics (ASDM), European wild boar (EUWB) and European domestics (EUDM), we found a marked correlation in allele frequencies between domestics and wild boars within Asia and within Europe, but not across continents, due to the large evolutive distance between pigs of both continents (~1.2 MYA). In general, the porcine species showed a small percentage of SNPs exclusive of each population group. EUWB and EUDM were predicted to harbor a larger fraction of potentially deleterious mutations, according to the SIFT algorithm, than Asian samples, perhaps a result of background selection being less effective due to a lower effective population size in Europe.

## Introduction

In this new era of sequencing, it is feasible to routinely obtain whole genome sequence data from an increasing number of individuals, making it possible the analysis of populations at the genomic level. The availability of this large amount of data allows us to study any species variability to an unprecedented detail. An intriguing observation from these studies is that, despite intensive selection and small effective sizes, animal domestic species harbor much more variability than anticipated [1–4]. In the particular case of the pig, this variability is especially remarkable and is likely caused by a complex demographic history and to the availability of a large amount of breeds [5–7].

The availability of a reference genome makes it feasible the large scale variant discovery with new sequencing technologies or 'next' generation sequencing (NGS). In pigs, the last assembly of porcine reference sequence (assembly 10.2) was released in November 2012 [8]. Although still incomplete, around 8% of the sequence is estimated to be missing from the assembly [8], it still constitutes the best resource to date in the genome of the pig. Currently, over a hundred pig sequences of about 20 different breeds and several countries have been published and are publicly available [8–12]. Despite these resources, so far, a comprehensive catalog of variants mined out these pig genomes is missing. Such a catalog is useful for many purposes: to design high density genotyping arrays, be it genome-wide or focused in specific genome regions or geographic origins of interest, to confirm SNPs from other experiments, to improve the reference genome, to identify variants of potentially large deleterious effect that can be followed up in functional studies, and to increase the general biological knowledge of a species. For instance, as we shall see, we discover a large mutational bias in the pig genome that is largely explained by the differential CpG content and recombination rate along the chromosomes.

Here, we report such a catalog (data have been submitted to dbSNP at the following URL: http://www.ncbi.nlm.nih.gov/SNP/snp_viewBatch.cgi?sbid=1062009 and they are also available at the website http://bioinformatics.cragenomica.es/numgenomics/), obtained from analyzing 128 genomes from wild boar and domestic pig samples worldwide distributed. In addition, we report annotation, allele frequencies in four major pig groups and we infer the ancestral allele for the majority of the SNPs. Knowledge of the ancestral allele is required for many statistical tests of selection so this is an additional value of the catalog here presented.

## Materials and Methods

### Samples

We analyzed a total of 133 suid genomes (S1 Table), 128 pigs (*Sus scrofa*) and five outgroups (*S*. *barbatus*, *S*. *cebifrons*, *S*. *verrucosus*, *S*. *celebensis*, and an African warthog, *Phacochoerus africanus*). The 128 pig genomes were classified into four large groups: Asian Wild Boars (ASWB, n = 41), that comprise wild boars from China, Japan and East Russia; Asian Domestics (ASDM, n = 23), including 9 Chinese breeds like Meishan or Xian; European Wild Boars (EUWB, n = 9) from Spain, France, Switzerland, and the Netherlands; and European Domestics (EUDM, n = 55) from all major breeds Duroc, Large White, Landrace, Pietrain Hampshire and local breeds (Iberian, Tamworth). European domestics include as well American village pigs, which have a predominant European, although hybrid, origin [13].

All samples had been shotgun sequenced with Illumina’s technology but at different depths, ranging from ~3× in a Tibetan wild boar [11] to 22× in a Wuzhishan pig, a miniature Chinese breed [9]. Here, we analyzed only two out of all available 25 lanes in the Wuzhishan pig so depths could be comparable across samples. The majority of genomes data were public [8,9,11,12] and 26 additional unpublished genomes were also used (E. Bianco *et al*., submitted). Main sample details are in S1 Table. In all, over 28 x 10^9^ reads, occupying around 2Tb of disk in bam format, were analyzed.

### Alignment and variant calling

The detailed bioinformatics pipeline is in S1 Script. The samples from Groenen *et al*., [8] (n = 50) were downloaded as bam files mapped against assembly 10.2. For the rest of samples, raw reads were mapped against assembly 10.2 with BWA [14] allowing for 7 mismatches and using default options otherwise. Duplicate removal and sorting were done with samtools v 0.1.18-sl61, using rmdup and sort options, respectively [15]. For all bam files, both the downloaded bam files and those generated in-house, GATK v. 2.7 IndelRealigner [16] was ran to improve the alignment around indels, default options were used.

Genotypes were called for each individual separately using samtools (v 0.1.19+) mpileup option and filtered with vcfutils.pl varFilter [15]. We excluded indels in this analysis because of their low reliability for the range of depths in our samples [17]. For a SNP to be called, we set the minimum depth to 5× and the maximum depth of twice the average sample’s depth plus one, minimum map quality and minimum base quality were both set to 20:

samtools mpileup-Q 20-q 20-m2-Dugf Sus_scrofa10.2.fas PIG_NAME.realigned.bam | bcftools view-vcg—| vcfutils.pl varFilter-d 5-D (MEAN_DEPTH*2)+1-Q 10 > PIG_NAME.iSAM.flt.vcf

Individual vcf files were then merged using custom scripts. For each individual, missing variant positions were coded according to bcftools output without the “-v” flag to avoid variant calling; confident homozygous-reference calls were coded as &rsquo;0/0' (homozygous for reference allele), and the position was marked as missing ‘./.’ otherwise.

VCFtools v0.1.12a [18] was used to filter the resulting multi individual vcf file, to extract outgroup genotypes, to analyze each of the four groups separately, and to filter out genotypes for which raw depth was ≥ 5 but where their high quality read depth was lower than 5 (--mindp). Allele frequency and allele count were calculated with the options—freq and—count, respectively, and transition / transversion rate was calculated in windows of 1Mb with the options—TsTv and—TsTv-summary. R version 3.0.2 was used to plot results [19].

### Ancestral allele determination

The variant calling and the merging steps were done including the five outgroup samples. An awk script was used to ascertain the ancestral allele, applying the following criteria:

The SNP must be biallelic where at least one of the alleles is the reference allele.
  A) The SNP must be present in at least two *Sus spp*. genomes, and be homozygous in all *Sus spp*. samples where the SNP is called;
  B) Otherwise, it must be present in *P*. *africanus* genome and be homozygous.
The ancestral allele must be either the reference or the alternative allele in *S*. *scrofa* (that is, a third allele must not be segregating).

In sites not complying with these conditions, the ancestral allele was considered as unknown.

### Exclusive variants and diagnostic SNPs

Population allele frequencies were obtained with VCFtools [18]. We defined an exclusive segregating variant as a site in which the derived allele is segregating only in the target group and it is not present in any of the remaining groups. Only those biallelic RA sites (R refers to the reference allele and A, to the alternative), and where all four groups had at least 50% of individuals with genotypes called were used. Shared and private alleles were plotted with gplots R library with venn package [20].

Joint site frequency spectrum between groups was also calculated. For each group, we selected the modal group size (the number of samples *n* where the highest number of variants was called in exactly *n* individuals). The count of derived alleles was performed and plotted with R package lattice (levelplot option, [21]).

### Genome context

We evaluated whether the number of SNPs per window and the transitions / transversion rate (Ts/Tv) correlates with genome features knowing to affect variability: GC content (%GC), CpG count, gene density and recombination rate. GC percentage and CpG count were calculated based on the *Sus scrofa* reference genome assembly 10.2 [8], and gene density was obtained as the percentage of the window sequence that is part of a gene according to *Sus scrofa* 2.75 GTF annotation. Genes overlapping two or more windows were discarded. We used the recombination rate (cM/Mb) from Tortereau *et al*., [22] with the same genome partitioning as in that work, in windows of ~1 Mb long. In addition, we computed percentage of missing genotypes per individual per window, averaged over individuals. To quantify the effect of each variable, we fitted the following linear models using the R function lm [19]:

N_snps = β0 + β1 Ts_Tv + β2 log(rec_rate) + β3 GC_percentage + β4 CpG_count + β5 Gene_Density + β6 missing_rate + e, [equation 1]

and

Ts_Tv = β0 + β1 N_snps + β2 log(rec_rate) + β3 GC_percentage + β4 CpG_count + β5 Gene_Density + β6 missing_rate+ e, [equation 2]

We used the logarithm transformation of recombination rate because the raw values were highly skewed.

### SNP annotation

All variants were annotated with Ensembl variant effect predictor (VeP) pipeline v76 [23] on Ensembl version 76 (using dbSNP *build* 140). Among the terms used in the annotation, we focused on stop gain and stop lost (sequence variants which cause a premature stop codon or the stop codon is changed resulting in an elongation of the protein), missense variants (non synonymous variants) and synonymous variants (a variant in a coding region that does not change the aminoacid). The definition of all terms used is available at http://www.ensembl.org/info/genome/variation/predicted_data.html. Variant annotation was performed both on the whole data set and by group. When there was more than one alternative allele, all possible alternatives were retained. The effect of the aminoacid changes was predicted using SIFT [24,25], a tool that tentatively predicts whether a missense variant affects protein function because of sequence homology and of the physical properties of amino acids. Default options were used.

### Simulation of the bioinformatics pipeline

In this study, we used NGS data from different sources. These data are noisy and highly unbalanced, with highly variable depth across samples (S1 Table). Moreover, the pipeline applied is complex and the properties of the SNP calling procedure are not necessarily known. As a fundamental caution when analyzing such a complex data, it is advisable to evaluate, even if approximately, the performance of the pipeline applied. Here, we evaluated how reliable are the SNPs called and estimated how many SNPs were retrieved out of all those actually segregating in the samples by simulation. To do this, we employed Pipeliner [26], with small modifications. Pipeliner seamless integrates several steps and softwares:

1. Simulates, with the coalescence, genome data reflecting as much as possible the population analyzed.
2. Maps simulated SNPs into a reference DNA sequence, this is done by replacing the reference base by an alternative base in the SNP position for each haplotype and produces a fasta file for each sequence; next, each individual genome is created by randomly choosing two sequences.
3. Simulates the sequencing process producing reads that mimic Illumina’s technology; we used ART (v. 1.5.1, [27]) to do so.
4. Runs BWA [14] to map the reads against the reference sequence.
5. Analyzes the output and reports several statistics of interest; among them:
  - Recovery: percentage of original genotypes that are correctly identified.
  - Sensitivity: percentage of callable genotypes, i.e. present in sites that pass the filters used, that are correctly identified.
  - False Discovery Rate (FDR): percentage of genotype calls performed that are incorrect

Pipeliner was fine tuned to duplicate as faithfully as possible the actual bioinformatics pipeline used to analyze the real data. First, to obtain the ‘real’ sequences in our sample, we simulated 256 sequences (128 diploid individuals) with MaCS [28], using the following structured population model:

NUMINDS = 128

EUDM = 55

ASDM = 23;

EUWB = 9

ASWB = 41

macs NUMINDS*2 100000-t 0.0005-r 0.0005-I 4 EUWB*2 EUDM*2 ASWB*2 ASDM*2-n 1 0.2-n 2 0.5-n 3 2.5-n 4 3-em 0.049 2 4 5-eM 0.06 0-ej 0.07 2 1-en 0.09 1 5-en 0.2 1 7-en 0.21 4 10-ej 0.08 3 4-ej 10 1 4

The command above simulates an older first split into two populations (Asia and Europe,-ej 10 1 4) and, a much more recent event, the split between domestic and wild populations in both continents (-ej 0.08 3 4 and-ej 0.07 2 1). This model is very similar to the model of [29]. Parameters were chosen such that estimates of nucleotide diversity were similar to those found in the real data. For the ART simulator [27], average depths were set for each individual as those empirically observed, ranging from 3x to 22× (S1 Table). As reference genome, we randomly chose one of the 'European' sequences, given that the assembly was derived from a Duroc specimen [8].

Finally, alignment, variant calling and merging were done following the pipeline used for real data, which resulted in a simulated multi individual vcf file. We used mstatspop v.0.998982beta [30] to evaluate the proportion of correctly called SNPs, the proportion of false SNPs and not identified SNPs. The whole process was repeated 100 times. Note that, despite we tried to faithfully represent the complexities of SNP calling for our specific set of samples, we ignored known difficulties in mapping due to structural variants or repetitive elements. The whole pipeline to do the simulation is in S1 Script.

## Results

### In silico evaluation of the bioinformatics pipeline

**Fig 1:**
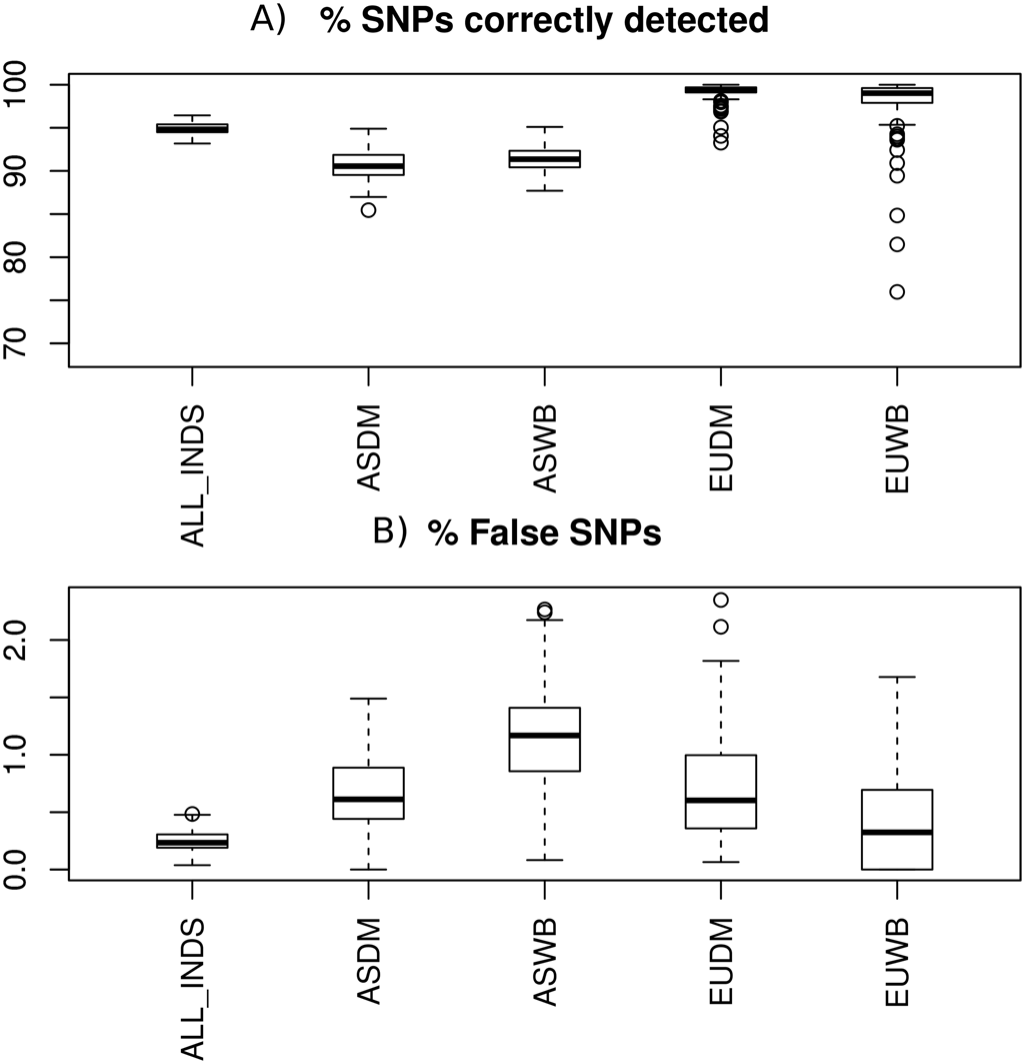
Using simulations, estimated percentage of segregating sites correctly detected (a) and percentage of false SNPs (b), according to the Pipeliner simulations. ALL_INDS: all samples; ASDM, Asian domestics; ASWB, Asian wild boar; EUDM, European domestics; EUWB, European wild boar.

**Fig 2:**
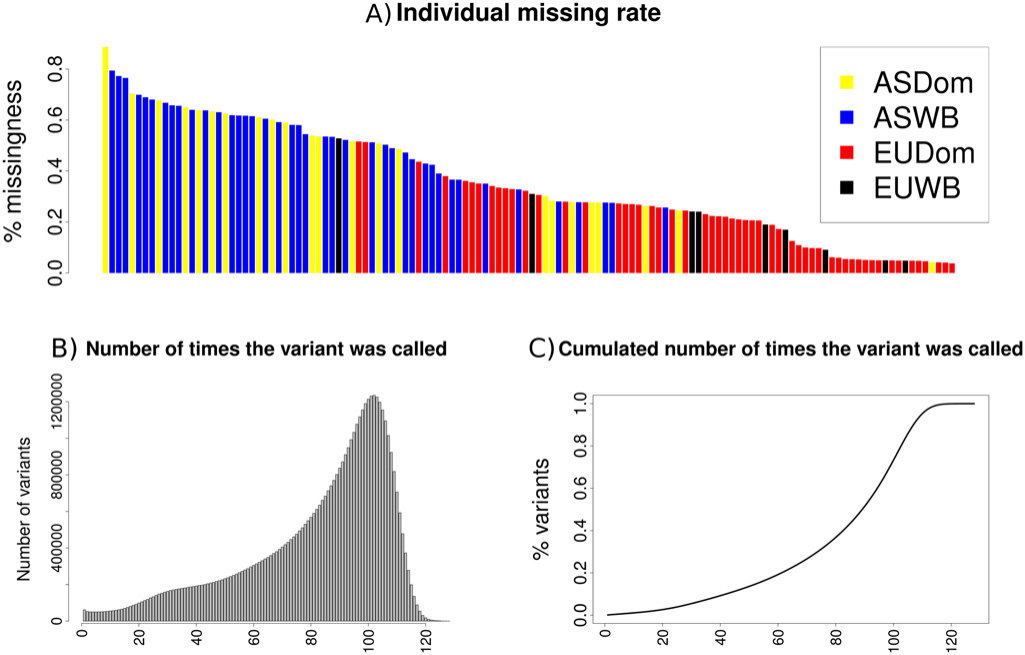
Individual missing rate (a); number of times a variant was called (b) and cumulative number of times a variant was called (c).

First, we evaluated our pipeline by simulation as described. We need to distinguish two issues. The first one is how many SNPs out of those segregating can be recovered. This is the main target in the real data analysis and, in this case, uniformly high depth and coverage may not be so critical because a SNP position that is not covered in one individual may have been covered in another one (provided is not a singleton). The second issue is how reliably called is each individual genotype. Accurate genotype calling is important for allele frequency estimates but not so much for SNP detection; for instance, suppose a heterozygous Reference/Alternative (RA) genotype is actually called as ‘AA’, the SNP will be equally identified, but frequency estimate will be strongly biased. With Pipeliner [26], we evaluated both issues as described in methods. Fig. 1 illustrates the overall expected power and percentage of wrongly identified SNPs. As can be seen, we expect to have discovered about 95% of all SNPs that may have been segregating in the 128 samples analyzed; of those, less than 0.5% variants are expected to be false positives. By population group, the outcome varies according to depth, the Asian populations being slightly worse than European populations because of shallower depth (Fig. 2). Even in those populations, power was 90% and FDR ~1%. The relatively high power of the pipeline, even at sallow depth, is due in part to the demographic model, which has very long branches between the Asian and European populations, followed by a bottleneck. This model, that reflects a plausible history of the pig genome, predicts an excess of non singletons compared to the neutral model; in turn, this means that a given SNP that is not called in one individual because of shallow depth can still be discovered in another sample. Singletons are unique and, therefore, this cannot happen in this case.

As for individual genotypes, we should expect according to the simulations, under the best case scenario, to recover ~70% of heterozygous genotypes, 73% of homozygotes for the alternative allele (AA) and 76% of the homozygotes for the reference allele (RR, S1a Fig). The average percentage of genotypes passing all quality filters that are correctly identified (sensitivity) is expected to be close to 1 for homozygote genotypes, and slightly lower for true RA genotypes (97.5%, S1b Fig). In all, the most likely reason for a SNP not to be correctly identified is that it was missed because of low depth or quality, rather than being incorrectly identified. FDR was very low, in the order of 1% for heterozygous genotypes (S1d,f Fig).

In all, Pipeliner simulations predict that we could expect our bioinformatics protocol to be highly reliable, allowing us to uncover about 95% of all SNPs in the samples with rather low false discovery rate. In practice, the real situation is likely to be worse than simulated because we are simulating the best case scenario, without considering the true complexities of the genome, e.g., duplications, indels, repetitive sequences, unequal GC content and so on. It is nevertheless difficult to consider all these complicating factors in a simulation, and Pipeliner results should be taken as an upper limit, mainly valid for well aligned genome regions.

### Individual missing and genotype rates

In the real data, we computed the number of identified SNPs in the whole sample that were not callable in each individual, as a percentage of all SNPs identified. The average individual SNP missing rate was 35%, ranging from 88% of an Asian Domestic (a Penzhou individual, [11]) to 4% of a European Domestic individual (an Iberian domestic, Bianco *et al*, submitted). Logically, missing rate was highly correlated with average depth: the lowest depth individuals, most of them ASDM and ASWB samples, had the highest missing rate (Fig. 2a). This high variability in missing rate is reflected in the number of times a SNP was genotyped in the dataset: 61,665 variants were genotyped in only one individual and only 23 SNPs were genotyped in all individuals (Fig. 2b).

The cumulative number of SNPs arranged by the times each SNP was called is in Fig. 2c: 50% of SNPs were called in 90 individuals and the SNPs genotyped in more than 115 individuals were less than 1% of the total variants counts. In other words, we found basically the same number of SNPs in 115 samples than in the whole set (n = 128).

### General SNP statistics and genomic context

**Fig 3:**
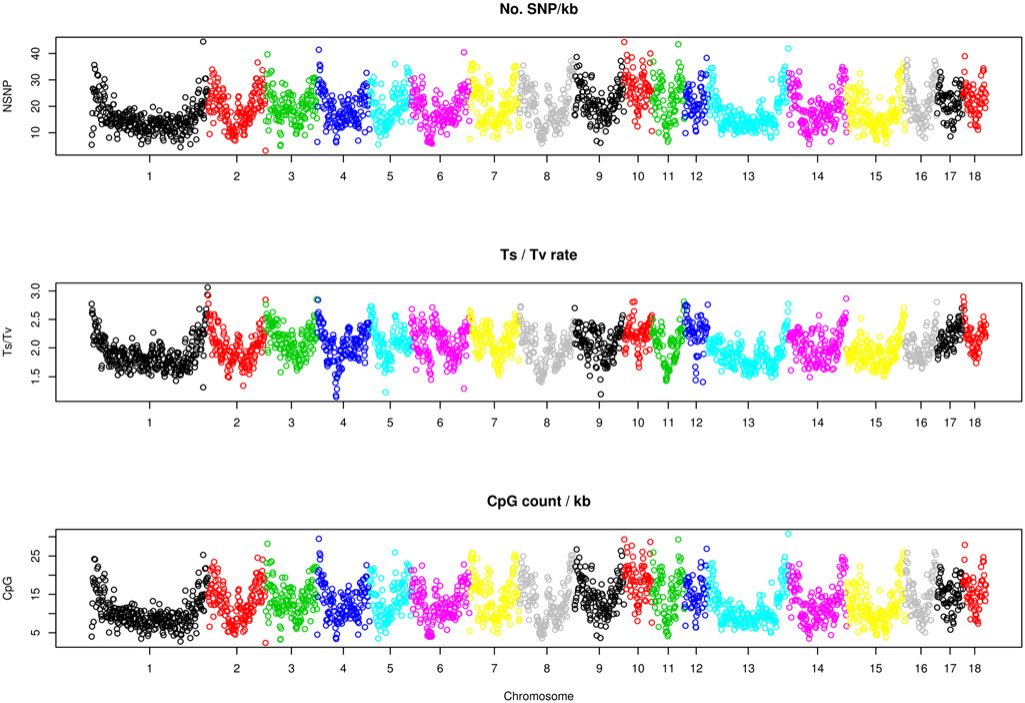
Number of SNPs per kb (top), average transition / transversion rate (middle) and CpG count per kb (bottom) per window. On the x axis, each dot represents a window of ~1Mb long. Different colors correspond to different chromosomes, from SSC1 to SSC18.

We found, among the 128 *S*. *scrofa* samples, a total of 48,119,476 SNPs that were called in at least one individual and passed all depth and quality filters. The majority (97.5%) were biallelic variants, whereas the rest presented 1, 3 or 4 alleles (Table 1). For 377,922 variants, only the alternative allele(s) were found; 12% of the 362,740 variants called as homozygote for the alternative allele were called in only one individual, but 71,081 (0.15% of total variants detected) were called in at least 64 individuals. These latter SNPs could reflect errors in the assembly. On average, we found ~19,000 ± 7,000 variants per Mb window (Fig. 3). By chromosomes, chromosome 10 had the highest number of variants per bp, with 26.7 variants per kb. The lowest number of variants per kb was found in chromosome 1 (15.9 variants per kb) (S2 Table).

**Table 1:** Total variants detected and number of variants per allele number at that locus.

Number of Allele(s) at position	Num. of positions	%
1 allele (AA)	362,740	0.75
2 alleles (all)	46,918,498	97.50
2 allele (R/A)	46,903,316	97.47
2 allele (A1/A2)	15,182	0.03
3 alleles (R/A1/A2)	828,854	1.72
4 alleles (R/A1/A2/A3)	9,384	0.02
Total number of variants	48,119,476	

R = Reference allele; A = Alternative allele; A1 = Alternative allele 1; A2 = Alternative allele 2; A3 = Alternative allele 3.

Average transition / transversion rate was Ts/Tv = 2.04 ± 0.28. This average rate is comparable to that found in other species [31–33]. A higher than one Ts/Tv is expected because of the molecular mechanisms behind transitions and transversion but there was, nonetheless, a genomewide large variability (Fig. 3, middle); 125 windows showed Ts/Tv > 2.5 and 30 had Ts/Tv < 1.5, also see S2 Fig. Although mutational bias is known to vary widely along the genome, there was a striking apparent correlation between number of SNPs and transition / transversion rate, both increasing in telomere regions; CpG count followed also similar patterns (Fig. 3).

**Fig 4:**
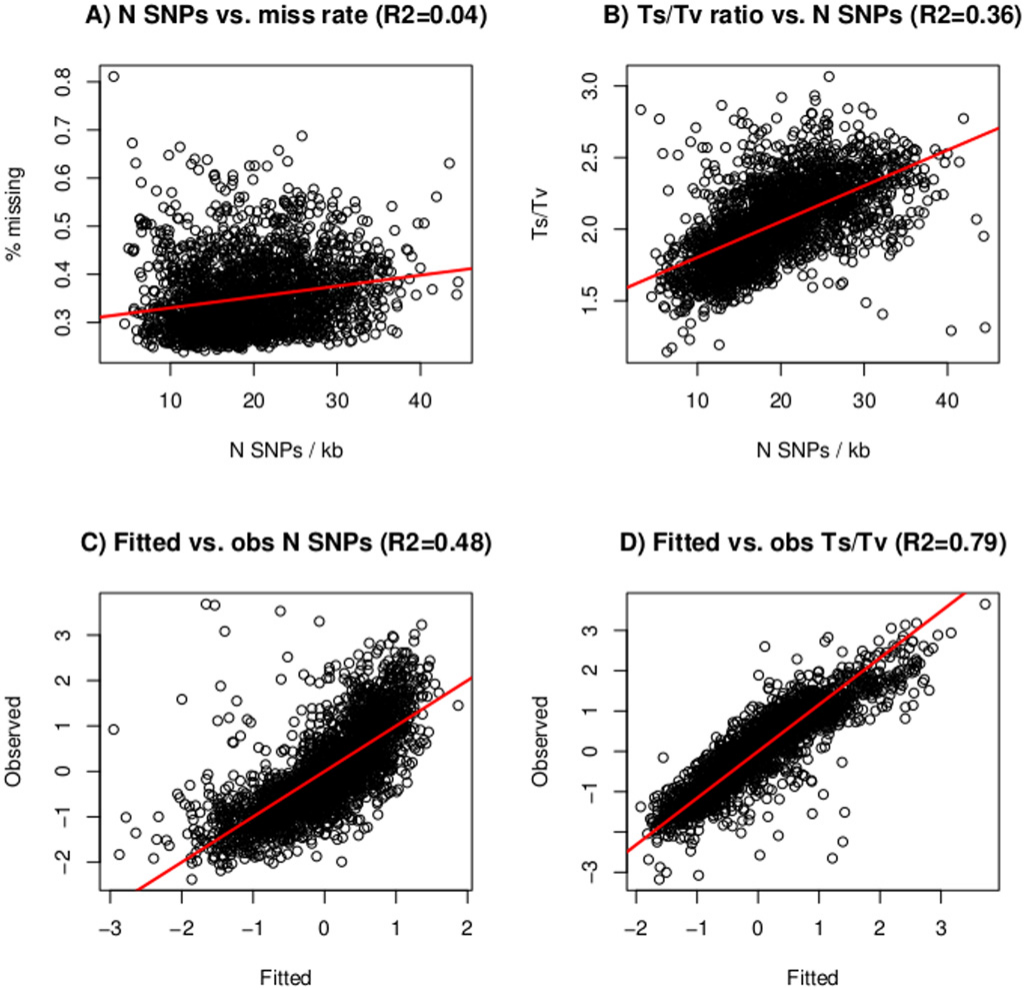
Number of SNPs vs. missing rate (a), and vs. Ts/Tv ratio (b); fitted vs. observed number of SNPs (c) and Ts/Tv (d) using equations 1 and 2, respectively.

We were intrigued by this observation, which seems not to have been reported previously. First, we noted that the rate of missing rate is correlated to the number of SNPs but this correlation was not too high, explaining only ~4% of variability for number of SNPs per window (Fig. 4a). Therefore, contrary to what would have been expected, missing rate is not relevant to predict the number of SNPs in a window or, in other words, this means that mapping alignment quality and depth (the two most influential factors in calling SNPs from NGS data) are independently distributed of nucleotide variability, at least in our data (Fig. 4a). In contrast, a much stronger relation was found between number of SNPs and Ts/Tv rate genomewide (R^2^ = 0.36, Fig. 4b).

In an attempt to explain how number of SNPs and Ts/Tv are interrelated, we fitted the linear models in equations 1 and 2. Table 2 shows the estimates of each independent variable, in increasing order of fit explained (R^2^). For number of SNPs, Ts/Tv rate and recombination rate suffice to explain most of variability, whereas missing rate explains, marginally, only 3% increase in R^2^. This indicates that the increase in number of SNPs per window is partly explained by an increase in the number of transitions. Gene density in turn is almost irrelevant, in agreement with our previous results [10]. All variables together explain 52% of variability, which means that there are still many more factors than those studied here that are relevant for explaining the number of SNPs per window (Fig. 4c, Table 2).

**Table 2:** Multivariate regression estimates for number of SNPs and Ts/Tv ratio.

Number of SNPs / kb			
Independent variable	Estimate ± SD	t-statistics	Increase in R^2^
Ts/Tv rate	0.56 ± 0.03	19.59***	0.36
Log(rec. rate)	0.34 ± 0.02	18.20***	0.12
Missing rate	-3.12 ± 0.39	-7.90***	0.03
Gene density	-0.12 ± 0.12	-7.69***	0.01
GC %	0.11 ± 0.03	3.82***	<0.01
CpG count / kb	-0.03 ± 0.04	-0.61	<0.01
Sum			0.52

**Ts / Tv rate**			
Independent variable	Estimate ± SD	t-statistics	Increase in R^2^
CpG count / kb	0.62 ± 0.02	24.18***	0.61
Log(rec. rate)	0.24 ± 0.01	20.19***	0.14
N SNPs / kb	0.25 ± 0.03	19.59***	0.04
Missing rate	0.67 ± 0.27	2.53*	<0.01
GC %	-0.02 ± 0.02	-1.21	<0.01
Gene density	0.01 ± 0.02	1.14	<0.01
Sum			0.79

All dependent and independent variables are standardized, except percentage of missing values; recombination rates are log-transformed;

*, P<0.05;

***, P<10^-3^.

Note that variables tend to be significant even if its effect is small because of the large number of observations (windows). For that reason, the increase in R^2^ due to each variable is a more useful assessment of its importance.

The results for the Ts/Tv ratio are far more interesting. First, most of variability (61% out of 79%) is explained by CpG count (Table 2); this is likely due to the high instability of methylated CpG sites, which frequently mutate towards transitions [34]. Further, differences in recombination rate explain another 14% in R^2^, whereas the rest of factors are only marginally relevant. Note that GC% per se, once corrected by the other factors, is not important, nor is gene density. In all, variability in Ts/Tv rates is fairly well explained (Fig. 4d) by a differential composition in CpG in the genome and varying recombination rates. Our analyses also suggest, but do not prove, that the correlation of number of SNPs and Ts/Tv ratio that we observe is likely an indirect consequence of both variables being affected by the same genome features, *i*.*e*., recombination rate and high mutability of CpG rich regions.

### Ancestral allele

The ancestral allele was determined for biallelic (RA only) and monoallelic (AA) SNPs. Following the rules detailed in methods, it was possible to determine the ancestral allele for 39,017,375 out of 47,266,056 such SNPs (82%). Of them, 31,939,953 (82%) had the reference allele as ancestral, whereas the opposite occurred in the remaining SNPs. The number of times the reference allele is expected to be the ancestral allele can be approximated by the frequency of the ancestral allele across SNPs in a population of size 2N, where N is the number of individuals analyzed. This frequency *q* can be obtained from Ewen’s sampling formula, $q_{N}=1/(\sum_{i=1}^{2N-1}1/i)$. Due to large variability in missing rate (Fig. 2a), the number *N* to choose is not clear. Taking *N* = 100 (the modal sample size, Fig. 2b), the expected frequency of the ancestral allele is 0.83 and for *N* = 128, *q* = 0.84, *i*.*e*., very close to what was observed (82%).

### Variants per population group

We calculated the number of variants per group (Table 3). The lowest number of variants was detected in European Wild Boars, which was also the group with fewest samples, whereas the highest number of variants was found in the Asian Wild Boars, also the most numerous group, although at lower average depth (S1 Table, Fig. 2a). Note that the expected number of SNPs (S) to be detected is proportional to the number of samples sequenced, in a neutral model, E(S) = *q*_*N*_ θ L, where *q*_*N*_ is Ewen’s sampling term, *q* is nucleotide diversity per base pair, and L, the length sequenced.

**Table 3:** Number of individuals and variants detected per group of populations.

Group	Number of individuals used	Number of Variants detected	Exclusive segregatingvariants	Exclusive fixed variants
ASDM	23	26,499,318	1,660,106	0
ASWB	41	35,719,205	8,089,523	0
EUDM	55	29,564,324	4,363,064	0
EUWB	9	12,562,569	915,532	14

Exclusive and fixed variants when filtering by SNPs called in at least 50% of the individuals in each group.

**Fig 5:**
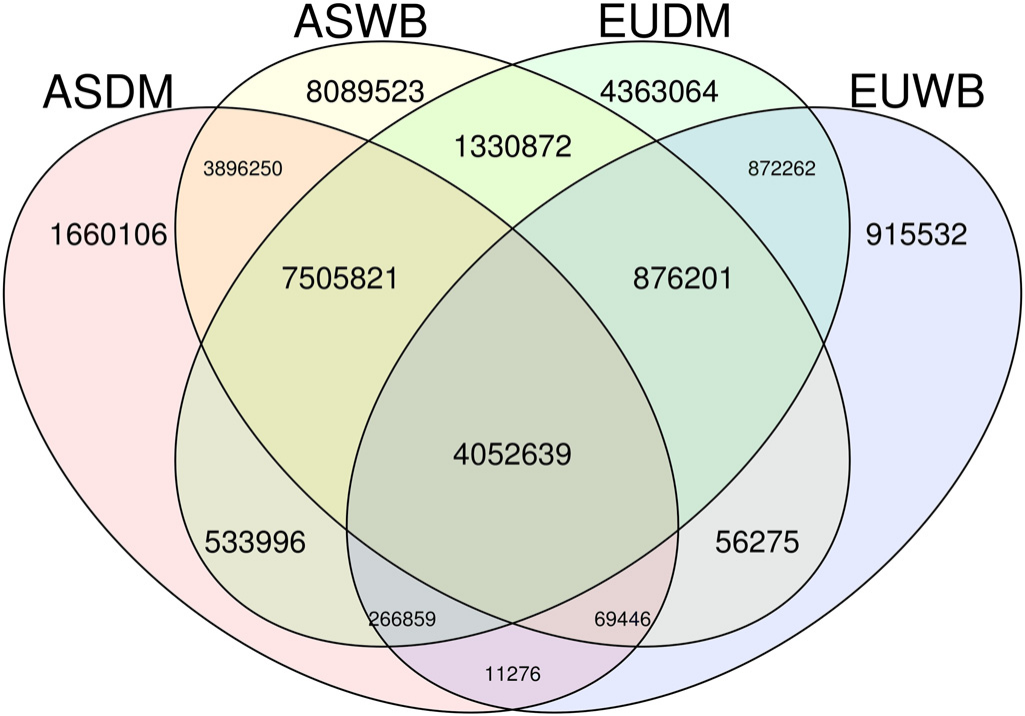
Number of exclusive and shared variants in the four groups. In each population, the variant must have been called for at least in 50% of the sample size.

Next, we investigated for how many SNPs the derived allele was specific to one pig group or shared between two or more groups. To do so, we used the 34,500,122 variants where the ancestral allele was identified and called in at least 50% of the pigs in each group. Fig. 5 shows the results in a Venn plot. A total of 4,052,639 (12%) variants was segregating in all four groups; in 39% of the variants (1,660,106 + 8,089,523 + 3,896,250), the derived allele was present only in Asian populations, whereas 18% (4,363,064 + 915,532 + 872,262) were exclusive of European populations. We found that ~ 9M SNPs were found exclusively in wild boars, whereas ~6.5 M variants were exclusive of domestics. Not unexpectedly, because of their higher variability compared to European wild boars, Asian wild boar had the highest number of unique variants (8,089,523 or 23.5% of the variants) and European wild boars, the lowest (only 915,532 or 2.65%). Note, however, that almost none of the SNPs had an exclusive allele fixed in any of the groups (only 14 in European wild boar, Table 3).

**Fig 6:**
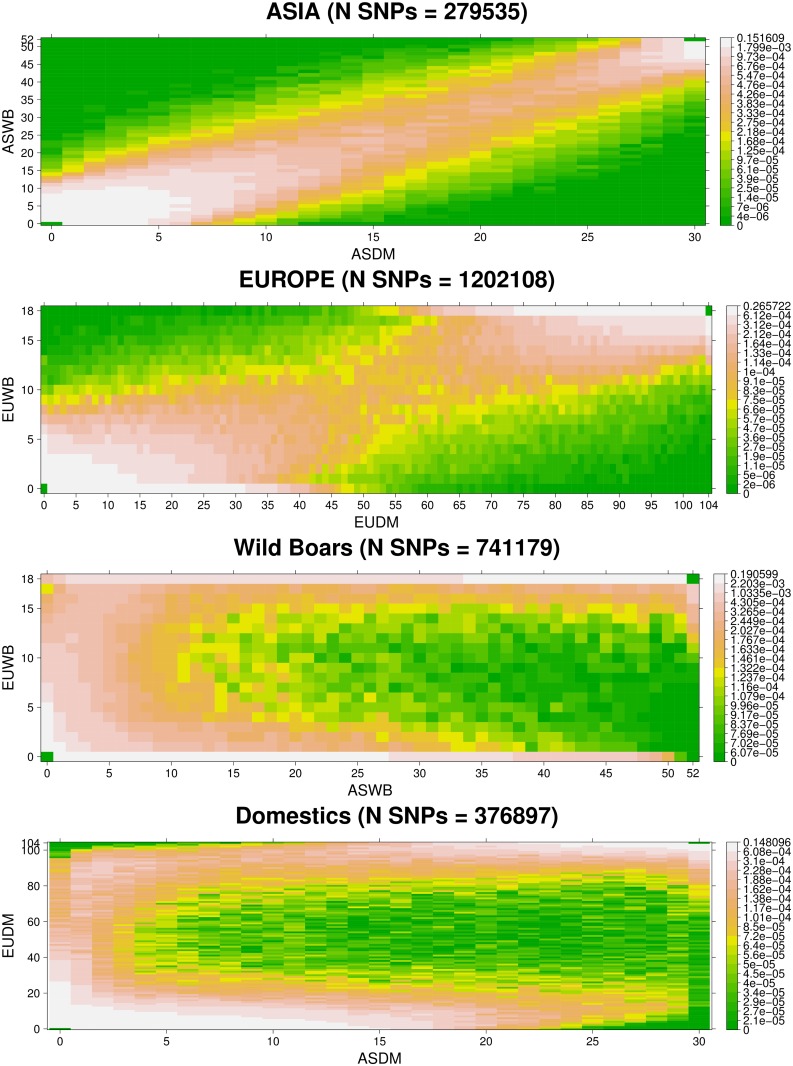
Joint site frequency spectra between population groups. Only SNPs found in the modal number of samples per groups were used. In each figure, x and y axis represent counts of the derived allele from 1 to 2N in each population, where N is the number of samples having the largest number of SNPs genotyped. Note that a count of 2N in say axis x means that the derived allele is fixed in that population but the same SNP can be segregating in the other population. The frequency of bivariate counts is represented in colors, with the log-scale as shown in the vertical bar. The more frequent a class is, the lighter the color, where dark green correspond to rare classes.

Joint site frequency spectra between populations, that is, how correlated are allele frequencies between populations, is a useful tool to infer demographic parameters (e.g., [35]). Here we computed the SNP joint site frequency spectrum between population groups: Domestics vs. Wild within continents and Asia vs. Europe within domestication status. Given that the number of individuals genotyped for each SNP varies, we only considered for these calculations the SNPs present in the modal group size, that is, for each group, the number of samples *n* that contained the maximum number of SNPs genotyped in exactly *n* samples (Fig. 6). Note that the spectra are rectangular due to unequal number of samples per group. Comparisons of wild boar vs. domestics within continents show a diagonal pattern, that is, a positive correlation in allele frequencies between wild boar and domestics; this is the outcome of domestics being derived from local wild boars in each continent. There are some interesting differences between Asia and Europe though. In Europe, the pattern is somewhat less marked and with an increased density of markers at extreme frequencies (very low and very high allele counts). We interpret this as the result of low effective population size in Europe and the marked divergence of Asia and European groups. In contrast, the joint spectrum between continents was completely different, a result of the long evolutionary distance that separates Asian from European pigs (> 1 MYA), be it wild or domestics. In this case, the joint spectrum is dominated by alleles at extreme frequencies, particularly in Europe. For instance, consider the lowest and uppermost rows in ASWB vs. EUWB, they correspond to SNPs that may segregate at intermediate frequencies in Asia, but are singletons in Europe, and make most of the SNPs. This pattern is also observed when contrasting ASDM vs. EUDM although less marked, likely a result of Asian introgression in EUDM.

### Variant annotation

The 48,119,476 variants called were analyzed by the VeP program (v-76, [23]). We found SNPs overlapping with 21,455 out of the 25,322 annotated genes and 25,166 transcripts. About half (22,336,270) of the variants were novel, *i*.*e*., not present in dbSNP (*build* 140), variants. Most of the SNPs were annotated into intergenic (67.5%) or intronic (29.5%) regions, about 1% (463,030 SNPs) were annotated into coding regions. On average we found 1,013 SNPs per gene, including coding and non coding variants, as well as upstream, downstream regions and intronic variants. The detailed number of variants per category and per chromosome is in Supplementary material S3 Table. Among the 463,030 SNPs annotated into coding regions, 246,976 were synonymous. The most severe variant classes, according to their predicted functional consequences, are listed in Table 4 for all individuals and by group.

**Table 4:** Summary of the SNP annotation results for the most deleterious consequence obtained using VEP.

	ALL	ASDM	ASWB	EUDM	EUWB
Splice donor variant	1,325	452	538	898	297
Splice acceptor variant	1,280	391	542	887	257
Stop gained	5,224	873	1,373	3,630	739
Stop lost	174	74	98	124	44
Initiator codon variant	359	155	209	237	98
Missense (non-synoynmous) variant	168,785	61,401	83,093	110,565	39,205
Splice Region variant	38,562	18,541	23,852	24,432	9,410
No. potentially deleterious (SIFT<0.05)	48,379	12,918	19,303	30,087	9,011
Total gene variants	21,741,159	10,732,921	14,632,536	12,645,639	4,941,666
Intergenic	33,988,809	17,284,550	23,626,744	21,137,970	7,829,133
Total variants*	48,119,476	25,427,907	34,471,527	29,073,261	11,602,230

Annotation term order is in decreasing order of severity according to Ensembl ^#^.

*Note that the total of intergenic + genic variants is greater than the number of variants because it includes all those variants carrying more than one allele.

^#^ **(http://www.ensembl.org/info/genome/variation/predicted_data.html)**

Stop gains overlapped 3,502 genes. Of them, 30 had the stop codon as homozygous in all samples (*i*.*e*., the reference allele was not found). The 174 stop lost SNPs found were located in 160 genes, 10 of them had the alternative allele fixed, suggesting a longer protein than that annotated or an error in the annotation. A summary description of these genes is in S4 Table.

A total of 168,785 non-synonymous (missense) variants were found in 15,790 genes. SIFT predictions were obtained from 166,958 of these variants; 29% were predicted to have deleterious consequences (SIFT score < 0.05) on protein function (S3 Table). By population group, the percentage of predicted deleterious missense variants ranged from 12% (ASDM) to 28% (EUDM, S3 Table). We also identified how many SNPs with extreme frequency (>0.8 and <0.20) differences between wild boar and domestics were synonymous or non-synonymous. In contrast to Rubin *et al*., [36], we did not find any over representation of non synonymous variants in domestics, neither in Europe nor in Asia (S5 Table). The most likely reason for this discrepancy is that Rubin *et al*. [36] pooled Asian and European wild boars; if we repeat the analyses with the same wild boar pool as these authors, we also retrieved an excess of non synonymous mutations in domestic pigs. A further complication for this analysis is that sample size is quite unbalanced, especially in Europe, so the presence of a new SNP in EWB can largely sift the population allele frequency.

## Discussion

### The pig is a highly variable and diverse species

To our knowledge, we present the most comprehensive SNP catalog of any livestock species to date. Using primarily published sequences, we identified over 48 million variants in the autosomal pig genome, which is more than the 28.3M SNPs recently reported in cattle [4]. Despite unequal and sometimes shallow coverage, the number of SNPs discovered per Mb was ~19,000 or one per 50 bp. This clearly shows how massive sequencing efforts have the ability to unfold vast amounts of hidden variability that could not have been detected until now. This work therefore expands dramatically the catalog of variants that are of potential interest in the pig breeding industry and beyond, given that the pig is also an important biomedical model. This effort, it should be noted, refers only to SNPs, similar works remain to be done for structural variants, mainly CNVs and indels.

Of the 48M identified SNPs, 46% were novel, indicating as well how incomplete are the porcine genomic resources available so far. These SNPs overlapped with 21,455 out of the 25,322 pig annotated genes, and we found an average of 1,013 variants per gene. Further, this catalog was obtained from worldwide samples, domestic and wild, making it an unbiased account of polymorphism in the species. Simulations suggest that the dataset generated should be highly reliable, at least for non complex regions where read mapping is not an issue. Simulation of the NGS pipeline with Pipeliner [26], using exactly the same options and comparable depth for each of the 128 pig samples, suggest that the SNPs reported are very likely to be real (FDR ~ 1%) and that we have uncovered a large percentage of the SNPs segregating in the populations sequenced. We estimate that, in the best case scenario, excluding NGS mapping problems in complex genome regions, about 90% of the SNPs segregating in the Asian samples sequenced and close to 100% for European samples sequenced may have been detected (Fig. 1). At least for genome regions with good mapping properties, we have likely reported a large part of common SNPs in the pig species. Aside from genome complexities, It should be noted that, in current assembly, still about 8% of genome is estimated to be missing [8] so using an improved future assembly could even increase the amount of SNPs that can be retrieved from the same dataset.

### Genomic context does matter

As in Drosophila and other species, including pigs [10,37,38], we found a significant correlation between recombination rate and number of polymorphisms, as predicted by models of hitchhiking and background selection [39,40]. In general, we also found an increased number of SNPs towards telomeric regions (Fig. 3).

But perhaps the most surprising observation is that this increased number of SNPs is largely explained by a correlated change in mutational bias Ts/Tv, and not by the percentage of missing values caused by shallow depth (Fig. 3, Fig. 4b, Table 3). In turn, most of this Ts/Tv bias is explained by CpG content and recombination rate (Fig. 4d). We are not aware of this phenomenon having been reported in other species, and whether this happens in other mammal or non mammal species should be investigated further. Our analyses suggest that an elevated CpG content subsequently increases the ratio of transition/transversion caused by methylation and affecting, indirectly, the number of SNPs.

### The pig species has relatively few group exclusive SNPs

Ascertaining SNPs with extreme frequencies between groups is useful for traceability purposes, and to identify signatures of selection and of domestication. We looked for exclusive variants in all pairwise comparisons (ASDM, ASWB, EUDM, and EUWB), and also between domestics and wild boar between and within continents, setting the minimum sample size to half the group size per each group. About 4M SNPs were segregating in all groups, suggesting that these SNPs are very old, prior to divergence between the European and Asian clades that occurred ca 1 MYA or that they were introgressed more recently in European breeds from Asia [41]. Asian wild boar showed the highest number of private variants (> 8 million, Fig. 5), in agreement with an Asian origin of the species [6,42], and the larger geographic span of sampling locations in Asia than in Europe. In contrast, less than 1M were exclusive of European wild boar. Interestingly, there were ~10 times more shared SNPs between domestics (ASDM vs. EUDM) than between wild boars (ASWB vs. EUWB). This could be due in part to the larger number of EUDM animals sequenced, but also to the introgression of Asiatic germplasm into European domestic breeds during late 18^th^ century onwards, which likely has introduced alleles that had been lost in the European wild boar [43,44].

### Higher frequency of potentially deleterious variants in Europe than in Asia

Annotation is one of the most critical and time consuming aspects of any genome, and that of the pig is still largely based on *in silico* automatized procedures. Therefore, the SNPs annotation provided here cannot be considered the definitive annotation; furthermore, about 8% of the pig genome is thought to be missing from current annotation [8] so these results should be taken with some caution. Similarly, a low SIFT [25] score cannot be taken as an infallible proof of damaging status because these algorithms are error prone and also, SIFT is based on the premise that function and protein evolution are correlated, and rely on protein conservation though species [24]. Nevertheless, they can serve as guide to prioritize variants that can be of interest for follow up studies.

With all these caveats in mind, it is nonetheless interesting to remark that we found a higher proportion of potentially deleterious variants in European Domestics (24%) and European wild boars (18%) than in Asian pigs (12%, Table 4). Although further works to verify this should be done, it could be due to the lower effective population size in European populations, as compared to Asia, which in turn results in natural selection being less effective in purging deleterious alleles. An alternative explanation would be that artificial selection in European breeds has resulted in an increase in alleles that are perceived as deleterious by current SIFT algorithms. However, this does not explain the increased frequency of potentially deleterious alleles in European wild boar.

## Conclusions

We have carried out a large scale data mining effort of currently available pig genomes to uncover over 48M autosomal SNPs; a parallel simulation study suggests that false discovery rate should be very low, at least in genome regions with good 'mappability'. About 40% of the SNPs had not been reported, which shows how incomplete pig genome resources are. Intriguingly, we have found a large variability in mutational bias (transition / transversion rate) along the pig genome that is primarily explained by differences in CpG content and recombination rate. As for number of SNPs per kb, it is relatively insensitive to the rate of missing values and it depends mainly on Ts/Tv and recombination rates. The pig is a species with a very complex demographic history, where European and Asian branches isolated ~1 MYA only to be crossed in modern times to result in the most widely used pig breeds worldwide. As a result, there exists a relatively small percentage of SNPs that are exclusive of these European breeds compared to other populations. In contrast, the differences between Asian and European wild boars are much higher.

### Ethics statement

DNA samples and genome analyses from all samples in this work have been published previously, and we refer to the original works for details [8–10,12,13,36,37]. DNA samples were obtained from blood samples collected according to national legislation, from tissue samples from animals obtained from the slaughterhouse, or from semen. For Spanish samples in particular, animal manipulations were performed according to the Spanish Policy for Animal Protection RD1201/05, which meets the European Union Directive 86/609 about the protection of animals used in experimentation.

## Supporting Information

S1 Script Scripts used in the analyses of the real data and to simulate the performance of the pipeline. (SH)

S1 Fig Simulated evaluation of expected genotype recovery (a) and sensitivity (b), error types for heterozygous genotypes (c), heterozygous genotype false discovery rate (d), error types in homozygous for the alternative allele (e), false discovery rate for homozygote alternatives alleles (f). RR = genotyped as homozygous for the reference; AA = genotyped as homozygous for the alternative; RA = genotyped as heterozygous.
(TIFF)

S2 Fig Genomewide distribution of standardized statistics by windows of ~1Mb. N SNPs, total number of SNPs per window; Ts/Tv, transition / transversion rate; CpG, number of CpG counts; log rec. rate, logarithm of recombination rate in cM/Mb from Tortereau et al.
(TIFF)

S1 Table Details of samples analyzed. ASDM, Asian Domestics; ASWB, Asian Wild Boar; EUDM, European Domestics; EUWB, European Wild Boar; N, number of samples; Average depth is calculated after filtering by base and map quality
(DOCX)

S2 Table Number and kind of variants detected per chromosome. (DOCX)

S3 Table Number of SNPs per annotation class and SIFT score of biallelic SNPs per population group. (XLSX)

S4 Table The ten genes where the premature stop codon mutation was fixed, together with the SNP location .
(DOCX)

S5 Table Derived nucleotide substitutions showing marked allele frequency differences between wild boars and domestic pigs. (DOCX)
