# Supplementary figures and images for: A Deep Catalog of Autosomal Single Nucleotide Variation in the Pig
Source: PLoS One. 2015 Mar 19;10(3):e0118867. doi: 10.1371/journal.pone.0118867 (PMC4366260; doi:10.1371/journal.pone.0118867)

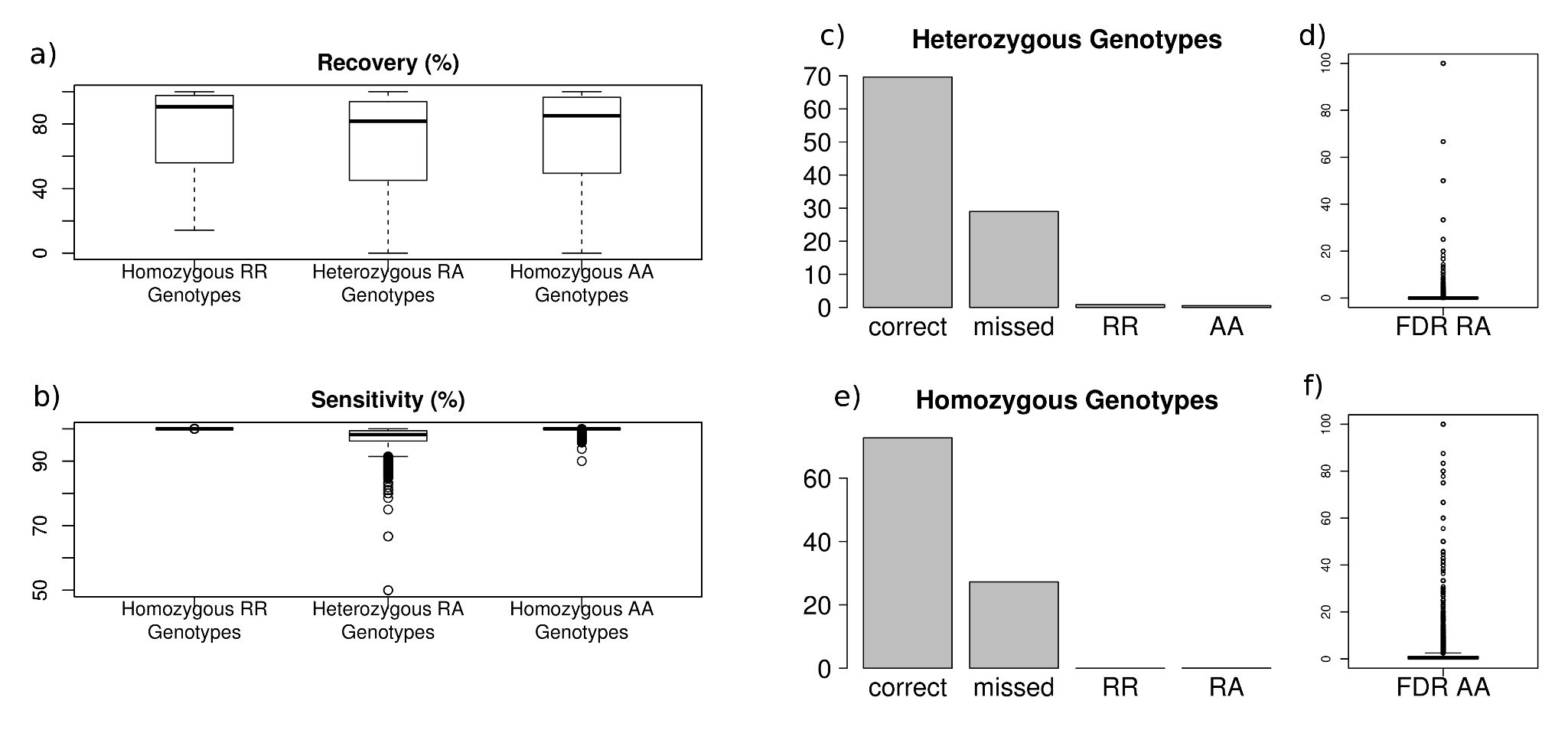

Supplement: S1 Fig — RR = genotyped as homozygous for the reference; AA = genotyped as homozygous for the alternative; RA = genotyped as heterozygous. (TIFF) [file pone.0118867.s002.tiff]

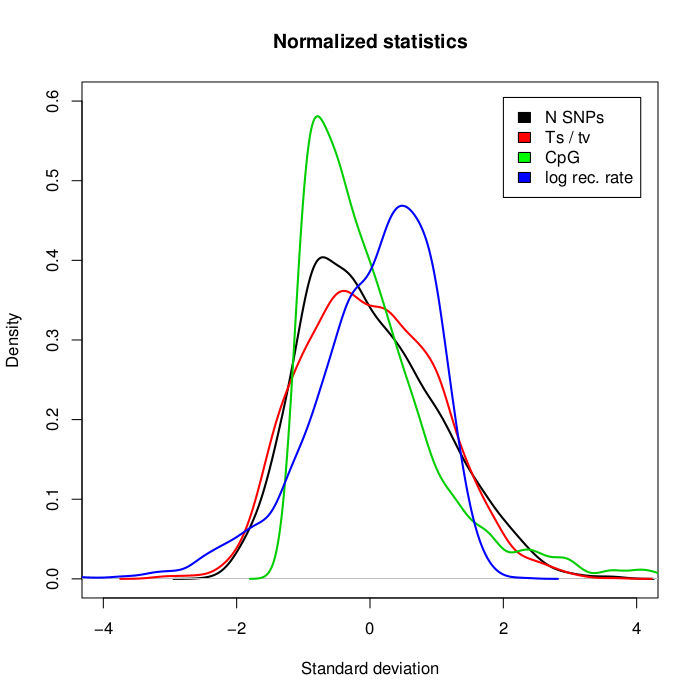

Supplement: S2 Fig — N SNPs, total number of SNPs per window; Ts/Tv, transition / transversion rate; CpG, number of CpG counts; log rec. rate, logarithm of recombination rate in cM/Mb from Tortereau et al. (TIFF) [file pone.0118867.s003.tiff]
