# Supplementary material for: A Deep Catalog of Autosomal Single Nucleotide Variation in the Pig
Source: PLoS One. 2015 Mar 19;10(3):e0118867. doi: 10.1371/journal.pone.0118867 (PMC4366260; doi:10.1371/journal.pone.0118867)
Supplement: S1 Table — ASDM, Asian Domestics; ASWB, Asian Wild Boar; EUDM, European Domestics; EUWB, European Wild Boar; N, number of samples; Average depth is calculated after filtering by base and map quality (DOCX) [file pone.0118867.s004.docx]

Table S1: Details of samples analyzed.

| **Group** | **Accession Number** | **Breed** | **N** | **Average depth** | **Individual mean depth**  **range** | **Reference** |
| --- | --- | --- | --- | --- | --- | --- |
| ASDM | SRA065458 | Jinhua | 3 | 4.5 | (4.1-4.8) | Li  *et al.*, 2013 |
| ASDM | ERP001813 | Jiangquhai | 1 | 10.4 |  | Groenen *et al.*, 2012 |
| ASDM | ERP001813 | Meishan | 4 | 9.3 | (8.8-10.1) | Groenen *et al.*, 2012 |
| ASDM | SRA065458 | Neijiang | 3 | 5.3 | (4.5-6.1) | Li  *et al.*, 2013 |
| ASDM | SRA065458 | Penzhou | 3 | 4.2 | (3-5.1) | Li  *et al.*, 2013 |
| ASDM | SRA065458 | Wujin | 3 | 5.1 | (4.5-5.6) | Li  *et al.*, 2013 |
| ASDM | AJKK00000000 | Wuzhishan | 1 | 22.2 |  | Fang *et al.*, 2012 |
| ASDM | ERP001813 | Xiang | 2 | 9 | (8.9-9.1) | Groenen *et al.*, 2012 |
| ASDM | SRA065458 | Ya'nan | 3 | 4.6 | (4.3-5.1) | Li  *et al.*, 2013 |
| ASWB | ERP001813 | Sumatra | 2 | 11 | (10.8-11.1) | Groenen *et al.*, 2012 |
| ASWB | SRA065461 | Tibetan Wild Boar | 30 | 5.1 | (3.6-6.9) | Li  *et al.*, 2013 |
| ASWB | ERP001813 | Japanese Wild Boar | 1 | 11 |  | Groenen *et al.*, 2012 |
| ASWB | ERP001813; SRA065461 | Chinese Wild Boar | 7 | 6.4 | (4.2-10.1) | Groenen *et al.*, 2012; Li *et al.*, 2013 |
| ASWB | unpub. | Russian Wild Boar | 1 | 6.9 |  | Unpub. |
| EUDM | SRP044261; unpub. | Creole village pigs | 17 | 11.9 | (6.7-14.5) | Ramirez *et al.*, 2014; unpub. |
| EUDM | ERP001813 | Duroc | 4 | 9 | (6.1-11.6) | Groenen *et al.*, 2012 |
| EUDM | ERP001813 | Hampshire | 2 | 10.8 | (10.1-11.5) | Groenen *et al.*, 2012 |
| EUDM | SRP044261; unpub. | Iberian | 6 | 13 | (12.4-14.5) | Ramirez et al., 2014; unpub. |
| EUDM | ERP001813 | Landrace | 5 | 9.4 | (7.4-13.7) | Groenen *et al.*, 2012 |
| EUDM | ERP001813; unpub. | Large White | 16 | 9.5 | (5.8-12) | Groenen *et al.*, 2012; unpub. |
| EUDM | ERP001813 | Pietrain | 5 | 9.3 | (5.8-11.2) | Groenen *et al.*, 2012 |
| EUDM | unpub. | Tamworth | 1 | 13.3 |  | unpub. |
| EUWB | ERP001813; SRP044261; unpub. | European Wild Boar | 9 | 10.7 | (5.6-14.4) | Groenen *et al.*, 2012; Ramirez *et al.*; unpub. |
| Outgroup | ERP001813 | *Sus barbatus* | 1 | 7.1 |  | Groenen *et al.*, 2012 |
| Outgroup | ERP001813 | *Sus cebifrons* | 1 | 9.8 |  | Groenen *et al.*, 2012 |
| Outgroup | ERP001813 | *Sus celebensis* | 1 | 23.3 |  | Groenen *et al.*, 2012 |
| Outgroup | ERP001813 | *Sus verrucosus* | 1 | 12.5 |  | Groenen *et al.*, 2012 |
| Outgroup | ERP001813 | *Phacochoerus africanus* | 1 | 12.7 |  | Groenen *et al.*, 2012 |

ASDM, Asian Domestics; ASWB, Asian Wild Boar; EUDM, European Domestics; EUWB, European Wild Boar; N, number of samples; Average depth is calculated after filtering by base and map quality
