# Supplementary material for: A Deep Catalog of Autosomal Single Nucleotide Variation in the Pig
Source: PLoS One. 2015 Mar 19;10(3):e0118867. doi: 10.1371/journal.pone.0118867 (PMC4366260; doi:10.1371/journal.pone.0118867)
Supplement: S2 Table — (DOCX) [file pone.0118867.s005.docx]

Table S2: Number and kind of variants detected per chromosome

|  | **Size (bp)** | **Monoallelic** | **Biallelic** | **Biallelic R/A** | **Biallelic A1/A2** | **Triallelic** | **Tetraallelic** | **ALL VARIANTS** |
| --- | --- | --- | --- | --- | --- | --- | --- | --- |
| **SSC1** | 315,321,322 | 50,590 | 4,894,072 | 4,891,947 | 2,125 | 77,847 | 778 | 5,023,287 |
| **SSC2** | 162,569,375 | 20,174 | 3,191,651 | 3,190,846 | 805 | 57,243 | 607 | 3,269,675 |
| **SSC3** | 144,787,322 | 21,652 | 2,793,733 | 2,792,723 | 1,010 | 48,930 | 673 | 2,864,988 |
| **SSC4** | 143,465,943 | 22,839 | 2,788,682 | 2,787,696 | 986 | 47,783 | 461 | 2,859,765 |
| **SSC5** | 111,506,441 | 20,238 | 2,221,897 | 2,221,048 | 849 | 40,158 | 463 | 2,282,756 |
| **SSC6** | 157,765,593 | 22,664 | 2,895,610 | 2,894,704 | 906 | 51,856 | 884 | 2,971,014 |
| **SSC7** | 134,764,511 | 14,967 | 2,776,147 | 2,775,514 | 633 | 49,923 | 533 | 2,841,570 |
| **SSC8** | 148,491,826 | 19,984 | 2,869,342 | 2,868,462 | 880 | 52,508 | 571 | 2,942,405 |
| **SSC9** | 153,670,197 | 20,809 | 3,059,892 | 3,059,038 | 854 | 54,477 | 600 | 3,135,778 |
| **SSC10** | 79,102,373 | 12,414 | 2,054,129 | 2,053,616 | 513 | 41,524 | 456 | 2,108,523 |
| **SSC11** | 87,690,581 | 16,164 | 1,917,730 | 1,917,075 | 655 | 36,864 | 380 | 1,971,138 |
| **SSC12** | 63,588,571 | 10,564 | 1,352,744 | 1,352,287 | 457 | 25,892 | 402 | 1,389,602 |
| **SSC13** | 218,635,234 | 33,192 | 3,726,672 | 3,725,253 | 1,419 | 59,282 | 584 | 3,819,730 |
| **SSC14** | 153,851,969 | 18,624 | 2,896,925 | 2,896,198 | 727 | 50,214 | 505 | 2,966,268 |
| **SSC15** | 157,681,621 | 25,270 | 2,755,009 | 2,753,970 | 1,039 | 46,624 | 540 | 2,827,443 |
| **SSC16** | 86,898,991 | 10,765 | 1,845,174 | 1,844,727 | 447 | 34,734 | 389 | 1,891,062 |
| **SSC17** | 69,701,581 | 13,201 | 1,508,464 | 1,507,916 | 548 | 28,313 | 311 | 1,550,289 |
| **SSC18** | 61,220,071 | 8,629 | 1,370,625 | 1,370,296 | 329 | 24,682 | 247 | 1,404,183 |
