# Supplementary material for: A Deep Catalog of Autosomal Single Nucleotide Variation in the Pig
Source: PLoS One. 2015 Mar 19;10(3):e0118867. doi: 10.1371/journal.pone.0118867 (PMC4366260; doi:10.1371/journal.pone.0118867)
Supplement: S4 Table — . (DOCX) [file pone.0118867.s007.docx]

Table S4: The ten genes where the premature stop codon mutation was fixed, and the SNP location.

| **Ensembl Gene ID** | **Associated Gene Name** | **Chr.** | | | **Gene Start (bp)** | | **Gene End (bp)** | | **Stop lost variant position** | | **Codon change ^#^** | |
| --- | --- | --- | --- | --- | --- | --- | --- | --- | --- | --- | --- | --- |
| **ENSSSCG00000013388** | PDE3B* | 2 | | | 47,573,503 | | 47,619,007 | | 47,618,921 | | Tga/Gga | |
|  | Uncharacterized protein ortholog to Phosphodiesterase 3B, cGMP-inhibited. GO annotations include cGMP-inhibited cyclic-nucleotide phosphodiesterase activity and 3',5'-cyclic-nucleotide phosphodiesterase activity. | | | | | | | | | | | |
| **ENSSSCG00000026421** | PKD2L2 | 2 | | | 145,644,303 | | 145,652,786 | | 145,652,785 | | tAg/tGg | |
|  | Polycystic kidney disease 2-like 2. Diseases associated with PKD2L2 include kidney disease, and polycystic kidney disease. GO annotations include calcium channel activity and calcium ion binding. | | | | | | | | | | | |
| **ENSSSCG00000029125** | FAM13B | 2 | | | 145,684,311 | | 145,752,899 | | 145,684,313 | | Taa/Aaa | |
|  | Family with sequence similarity 13, member B. GO annotations related to this gene include GTPase activator activity. | | | | | | | | | | | |
| **ENSSSCG00000029541** | PSD2* | | 2 | 147,515,778 | | 147,546,602 | | 147,540,285 | | Tag/Aag | |  |
|  | Undescribed protein orthologue to pleckstrin and Sec7 domain containing 2. GO annotations related to this gene include phospholipid binding and ARF guanyl-nucleotide exchange factor activity. | | | | | | | | | | |  |
| **ENSSSCG00000000509** | ZFC3H1* | 5 | | | 38,344,644 | | 38,372,139 | | 38,344,646 | | Taa/Aaa | |
|  | Uncharacterized protein ortholog to Zinc finger, C3H1-type. | | | | | | | | | | | |
| **ENSSSCG00000026323** | FAM50B | 7 | | | 2,371,717 | | 2,372,697 | | 2,372,697 | | taA/taC | |
|  | Family with sequence similarity 50, member B. | | | | | | | | | | | |
| **ENSSSCG00000028225** | DICER1 | 7 | | | 123,586,576 | | 123,624,391 | | 123,586,577 | | tAg/tTg | |
|  | DICER1, ribonuclease type III. This protein is a ribonuclease and it is required by the RNA interference and small temporal RNA (stRNA) pathways to produce the active small RNA component that represses gene expression. | | | | | | | | | | | |
| **ENSSSCG00000008787** |  | 8 | | | 31,861,892 | | 31,953,968 | | 31,953,870 | | tgA/tgC | |
|  | Uncharacterized protein. | | | | | | | | | | | |
| **ENSSSCG00000017907** | OMC | 12 | | | 54,039,543 | | 54,042,605 | | 54,041,858 | | Tag/Aag | |
|  | Uncharacterized protein ortholog to SLC25A11: solute carrier family 25 member 11. Mithocondrial oxoglutarate/malate carrier. | | | | | | | | | | | |
| **ENSSSCG00000023504** | SLC23A2* | 17 | | | 14,843,106 | | 14,859,601 | | 14,855,224 | | Tag/Aag | |
|  | Uncharacterized protein ortholog to solute carrier family 23 , member 2. Ascorbic acid transporter. | | | | | | | | | | | |
|  |  |  | | |  | |  | |  | |  | |
| ^#^ The SNP is in capital letters, reference / alternative allele | | | | | | | | | | | | |
| * Orthologous associated gene name | | | | | | | | | | | | |
| Description from www.genecards.org and orthologous from www.ensemble.org | | | | | | | | | | | | |
