# Supplementary material for: A Deep Catalog of Autosomal Single Nucleotide Variation in the Pig
Source: PLoS One. 2015 Mar 19;10(3):e0118867. doi: 10.1371/journal.pone.0118867 (PMC4366260; doi:10.1371/journal.pone.0118867)
Supplement: S5 Table — (DOCX) [file pone.0118867.s008.docx]

**Table S5: Derived nucleotide substitutions showing marked allele frequency differences between wild boars and domestic pigs by continent**

|  |  | **Europe** | |  | **Asia** | |
| --- | --- | --- | --- | --- | --- | --- |
|  | **N** | **Non synonymous** | **Synonymous** | **N** | **Non synonymous** | **Synonymous** |
| **Wild Boar** | 9 | 14 | 19 | 41 | 0 | 0 |
| **Domestics** | 55 | 1 | 2 | 23 | 1 | 3 |

Values are numbers of derived mutations in which the frequency of the derived allele is >0.80 in the indicated population and <0.20 in the other.
